# Primary aldosteronism and obstructive sleep apnea: What do we know thus far?

**DOI:** 10.3389/fendo.2022.976979

**Published:** 2022-09-29

**Authors:** Huai Heng Loh, Norlela Sukor

**Affiliations:** ^1^ Department of Medicine, Faculty of Medicine, Universiti Kebangsaan Malaysia Medical Center (UKMMC), Kuala Lumpur, Malaysia; ^2^ Department of Medicine, Faculty of Medicine and Health Sciences, Universiti Malaysia Sarawak, Sarawak, Kota Samarahan, Malaysia

**Keywords:** renin, angiotensin, aldosterone, sleep disorders, obesity, hypertension, RAAS

## Abstract

Both primary aldosteronism and obstructive sleep apnea are well-known causes of hypertension and contribute to increased cardiovascular morbidity and mortality independently. However, the relationship between these two entities remains unclear, with studies demonstrating contradictory results. This review aims to collate and put into perspective current available research regarding the association between primary aldosteronism and obstructive sleep apnea. The relationship between these two entities, clinical characteristics, clinical implications, outcomes of treatment, potential causal links and mechanisms are hereby presented.

## Introduction

Primary aldosteronism (PA) is now recognized as one of the most common causes of secondary hypertension (1). Patients with PA were demonstrated to have higher risk of cerebrovascular and cardiovascular events, cardiovascular mortality and renal injuries compared to patients with essential hypertension, independent of blood pressure levels (2). Unfortunately, this disorder of the adrenal gland is substantially under-diagnosed (3). For decades, it is believed that the activation of renin-angiotensin-aldosterone system (RAAS) regulated the biosynthesis of aldosterone (4–7). There is now growing evidence that aldosterone secretion is not solely under RAAS regulation.

Obstructive sleep apnea (OSA) is a chronic and potentially life-threatening sleep-related breathing disorder caused by periodic narrowing and obstruction of upper airway during sleep, leading to repetitive apnea and hypopnea episodes (8). The prevalence has continued to rise over the years, mainly driven by an increase in prevalence of obesity, which is one of the major causes of OSA. It is another well-known risk factor for hypertension and is demonstrated to affect more than 80% of patients with resistant hypertension (9). In addition, OSA is associated with multiple long-term health complications, which include cardiovascular diseases and metabolic disorders (10).

Many studies have attempted to demonstrate a relationship between OSA and RAAS. Activation of RAAS, especially excess aldosterone, has been implicated to play a pathophysiological role in the relationship between OSA and hypertension, particularly resistant hypertension and PA (11). In fact, the Endocrine Society has identified OSA in the presence of hypertension as one of the groups with high prevalence of PA, and thus now recommends screening of PA among this cohort of patients (12).

In animal studies, episodic hypoxia led to elevated blood pressure which was prevented by renal artery denervation or treatment with angiotensin receptor blockers, suggesting a relationship between OSA and RAAS (13). Subsequently, this led to few human studies examining this relationship, which are illustrated below. Nevertheless, to date, the relationship between these two entities remains unclear. As both PA and OSA are known to contribute to increased cardiovascular morbidity and mortality, understanding the relationship between these two entities is essential in improving healthcare management of these patients by reducing cardiovascular-associated risks.

Hence, this review aimed to gather and put into perspective current available research regarding the association between PA and OSA. It focused on the relationship between these two entities, and presented evidence for their clinical characteristics with its implications, outcomes of treatment as well as the potential causal links and mechanisms.

## OSA in PA

### Prevalence

In a cohort of 207 patients with confirmed PA, 67.6% were found to have OSA (64.4% in White, 70.0% in Chinese), of which 27.1% were mild, 21.7% moderate and 18.8% severe (14). An almost similar prevalence of 55% was also observed in a retrospective analysis of 71 Japanese patients with PA (15). The prevalence of OSA seems to be much higher in PA than non-PA population (59.5% vs. 42.4%), albeit not significant (*p*=0.058) (16). This is probably due to the small sample size. Nevertheless, apnea hypopnea index (AHI) was demonstrated to be higher among patients with PA compared to those without PA (*p*=0.024) (16).

The prevalence of OSA in patients with confirmed PA is summarized in **Table 1**.

**Table 1:** Prevalence of OSA in PA.

Author	Number of patients with confirmed PA, n	Prevalence of confirmed OSA, n (%)	OSA severity, n (%)
Prejbisz 2013 (16)	32	19 (59.4)	NA
Wolley 2017 (17)	34	27 (79.4)	Mild 9 (33.3) Moderate 8 (29.6) Severe 10 (37.0)
Buffolo 2019 (14)	207	140 (67.6)	Mild 56 (40.0) Moderate 45 (32.1) Severe 39 (27.9)
Nakamura 2021 (15)	71	39 (54.9)	Mild 12 (30.8) Moderate 16 (41.0) Severe 11 (28.2)

PA, Primary aldosteronism.

OSA, Obstructive sleep apnea.

NA, Not available.

### Clinical characteristics

Patients with PA and OSA were more likely to be males, older, with larger neck circumference, more abdominal obesity, higher body mass index (BMI) and worse metabolic profile. These individuals have higher blood glucose and triglyceride levels with lower HDL-cholesterol concentrations (14–16). The elevated plasma aldosterone level was noted to be significantly correlated with OSA severity. However, this correlation was only observed among the White, but not in the Chinese and Japanese cohort (14, 15), despite Chinese PA population demonstrated a more severe phenotype of OSA compared to the White (14). The discrepancy observed could probably be attributed to differences in craniofacial anatomy, adiposity and salt intake between Asians and Caucasians (18–20).

### Outcomes of treatment

When patients with OSA and co-existent essential hypertension, resistant hypertension or PA were given mineralocorticoid receptor (MR) blockade for total duration of 8 weeks to 8 months, a significant reduction in AHI, hypoxic index and oxygen desaturation index was observed along with a decrease in body weight, neck circumference and blood pressure in all three groups of patients (17, 21, 22). Nevertheless, the reduction in AHI among patients with PA was not uniform and the difference seen could be confounded by difference in population studied, methodologies and sample sizes.

Compared to medical therapy, the effect of surgical treatment among patients with PA and OSA is less studied. Adrenalectomy performed among patients with co-existing PA and OSA led to reduction in AHI and neck circumference (17). Nevertheless, the small number of patients (n=7) in this study limited statistical significance and certainty. In a larger cohort of patients with PA and OSA (n=48), the probability of OSA reduced significantly (Berlin score 1.69 pre-operation vs 1.33 post-operation, *p*<0.001) after adrenalectomy (23). However, the absence of OSA confirmation with sleep study might have explained the non-significant difference on the reduction of OSA probability between the surgical and medical therapies.

### Possible mechanisms

Hyperaldosteronism may worsen the clinical course of OSA in patients with PA due to aldosterone-induced fluid accumulation in the neck (24). Aldosterone excess leads to salt and water retention in the distal tubules (25) causing rostral fluid shifts and para-pharyngeal edema. With the presence of neck tissue congestion, this increases upper airway resistance and subsequently collapse, thus worsening OSA (26, 27).

Besides, in rat models, infused aldosterone acted centrally to increase brain RAAS activity, oxidative stress, and sympathetic drive (28). This aldosterone-induced activation of central receptors may also lead to abnormal regulation of central breathing mechanisms, leading to deterioration of OSA.

Aldosterone excess has detrimental effects on β cell function leading to hyperglycemia. Hence it is commonly associated with metabolic dysregulation including type 2 diabetes (2, 29). Moreover, hyperaldosteronism is reported to induce insulin resistance by several other mechanisms such as autonomous cortisol secretion, impairment of glucose uptake into the liver, increment of hepatic glucose release, and enhancement of insulin-like growth factor-1 signaling (2). This echoes the results from Framingham Offspring study which demonstrated that aldosterone level is positively correlated with development of metabolic syndrome and increment of systolic blood pressure (30, 31). Patients with type 2 diabetes were reported to have an almost 50% increased risk in developing OSA compared to those without diabetes, especially among insulin-treated cohort, suggesting the role of insulin resistance in development of OSA (32, 33). This could be contributed by several mechanisms, including mixed apneic events seen in patients with type 2 diabetes (34), increased oxidative stress, autonomic dysfunction (35) and weight gain secondary to anti-diabetic medications (33).

Soluble plasma pro-renin receptors, which are specific receptors for both renin and pro-renin, were found to be significantly higher in male patients with OSA compared to age matched non-OSA male (36). This might explain the higher prevalence of OSA seen in male patients with PA. These receptor levels were also demonstrated to be positively correlated with severity of OSA, but not BMI, which further supports the association between OSA and RAAS (36).

MR antagonists augment diuresis and reduce leg-to-neck fluid redistribution. This leads to reduction in pharyngeal edema and upper airway resistance, causing improvement in OSA severity (14).

## PA in OSA

### Prevalence

Among 203 multi-ethnic cohort of patients diagnosed with OSA and hypertension, the prevalence of PA was reported to be 8.9% (11.8% in White, 5.9% in Chinese) (14). The authors concluded that this prevalence is not significantly different compared to the prevalence of PA observed in earlier studies (8.9% vs 5.9% in general hypertensive population and 11.2% from referral centers). However, in these earlier studies, OSA was not screened for in the subjects, which may explain the comparable prevalence. Furthermore, the prevalence of PA in OSA reported in this study needs to be interpreted with caution as the study was performed in different centers, using different confirmatory tests, different kits for aldosterone and renin, with lack of single scoring center, which could have biased the results.

In another study of 94 patients with moderate-to-severe OSA and hypertension, PA was confirmed in 21.3% compared to 8% of those without OSA (37). The prevalence of other metabolic disorders was noted to be high in this studied population (diabetes and impaired fasting glucose 90%, difficult-to-treat hypertension 70%, resistant hypertension 60%). These show that PA is a common part of multi-morbidity in patients with OSA, including diabetes and resistant hypertension (38).

The prevalence of PA in patients with confirmed OSA is presented in **Table 2**.

**Table 2:** Prevalence of PA in OSA.

Author	Number of patients with confirmed OSA, n	Prevalence of confirmed PA, n (%)	Subtype classification, n (%)
DiMurro 2010 (39)	53	18 (34.0)	APA 5 (27.8) IHA 13 (72.2)
Buffolo 2019 (14)	230	18 (8.9)	APA 7 (38.9) IHA 10 (55.6) Undetermined 1 (5.5)
Dobrowolski 2021 (37)	94	20 (21.3)	APA 7 (10.0) IHA 18 (90.0)

OSA, Obstructive sleep apnea.

PA, Primary aldosteronism.

APA, Aldosterone-producing adenoma

IHA, Idiopathic hyperaldosteronism.

### Clinical characteristics

Majority of patients with OSA who were subsequently diagnosed with PA presented with uncontrolled blood pressure ≥150/100mmHg, resistant hypertension or hypokalemia (14, 37). The frequency of PA in patients who presented only with OSA symptoms is low (1/18 and 4/20 respectively) (14, 37). Plasma aldosterone level in patients with OSA and metabolic syndrome was significantly higher compared to patients with OSA without metabolic syndrome, and this level was significantly related to AHI, waist circumference, triglyceride and HDL-cholesterol levels (11). Among patients with moderate-to-severe OSA and type 2 diabetes, plasma aldosterone, plasma renin and urinary aldosterone levels were higher compared to non-OSA patients with type 2 diabetes, although no correlation was found between AHI and the RAAS components in this cohort (40).

### Outcomes of treatment

The use of CPAP therapy, ranging from 1 week to 12 months, among different cohort of patients with OSA, *ie* presence of metabolic syndrome (11), normotension (41), essential (42–45) and resistant hypertension (46, 47), and type 2 diabetes (40), showed significant reduction in RAAS components. Several studies which demonstrated lack of reduction in the RAAS components were mostly limited by a small sample size or short duration of CPAP use (48–53). To date, there are no studies evaluating the effect of CPAP therapy on RAAS components amongst OSA and PA patients.

### Possible mechanisms

Intermittent hypoxia was shown to increase plasma levels of renin and aldosterone. It also enhanced angiotensin (Ang) I expression and resulted in AngII stimulation of carotid body receptors in animal models (13, 54–57). Similarly, sleep fragmentation and repetitive arousals in patients with OSA may lead to activation of the RAAS, causing an increased secretion of AngI, AngII and subsequently aldosterone.

In animal studies, acute hypercapnia or hypoxia separately increased plasma aldosterone levels, which was independent of increases in plasma renin activity, suggesting a renin-independent pathway in aldosterone secretion (58, 59). Sleep fragmentation and repeated arousal induce stress which stimulates the release of ACTH from the pituitary (11). The persistent activation of sympathetic nerve during both sleep and wakefulness not only stimulates the RAAS (53), but also the hypothalamic-pituitary-adrenal axis in releasing cortisol (40). Both RAAS and ACTH synergistically regulate aldosterone pulse wave. While RAAS plays a major role at night when plasma cortisol concentration is low, elevated cortisol concentration controls the pulse amplitude of aldosterone during the daytime (40).

OSA is commonly found in patients who are obese. The adipose tissue present in obesity is an important source of RAAS hormone secretion, which is independent from the classical RAAS activation (60, 61). This is demonstrated in experimental studies which showed that adipocytes release adipokines and free fatty acids that could stimulate aldosterone secretion from the adrenocortical cells (59, 62, 63). The finding of renin-binding protein gene in adipocytes, which acts as renin inhibitor, might be involved in modulation of renin activity. AngI and AngII receptors were obtained in rodent and human adipocytes with increased expression of AngI gene, especially in visceral adipocytes (64).

The angiotensin converting enzyme (ACE) is a vital enzyme in RAAS, playing a major role in development of cardiovascular diseases with I/D polymorphism of the ACE gene. Interaction between OSA and the ACE gene I/D polymorphism was significantly associated with presence of hypertension among Swedish patients (65) but not in the Turkish cohort (66). Furthermore, it is shown that Caucasian individuals with DD genotype are more prone to develop hypertension (66), in contrast to II genotype in Asians (67). Hence, ethnic differences in the genotype distribution for ACE gene I/D polymorphism may explain the differences seen in RAAS dysregulation in patients with OSA of different ethnicity.

As intermittent hypoxia is closely related to activation of RAAS, the resolution of intermittent hypoxia by CPAP may decrease the activity of RAAS leading to a reduction in aldosterone level (45). Additionally, CPAP improves ventilation, reduces sleep interruption, reduces sympathetic excitability, and increases insulin sensitivity, which in turn reduces aldosterone level (40).

**Figure 1:**
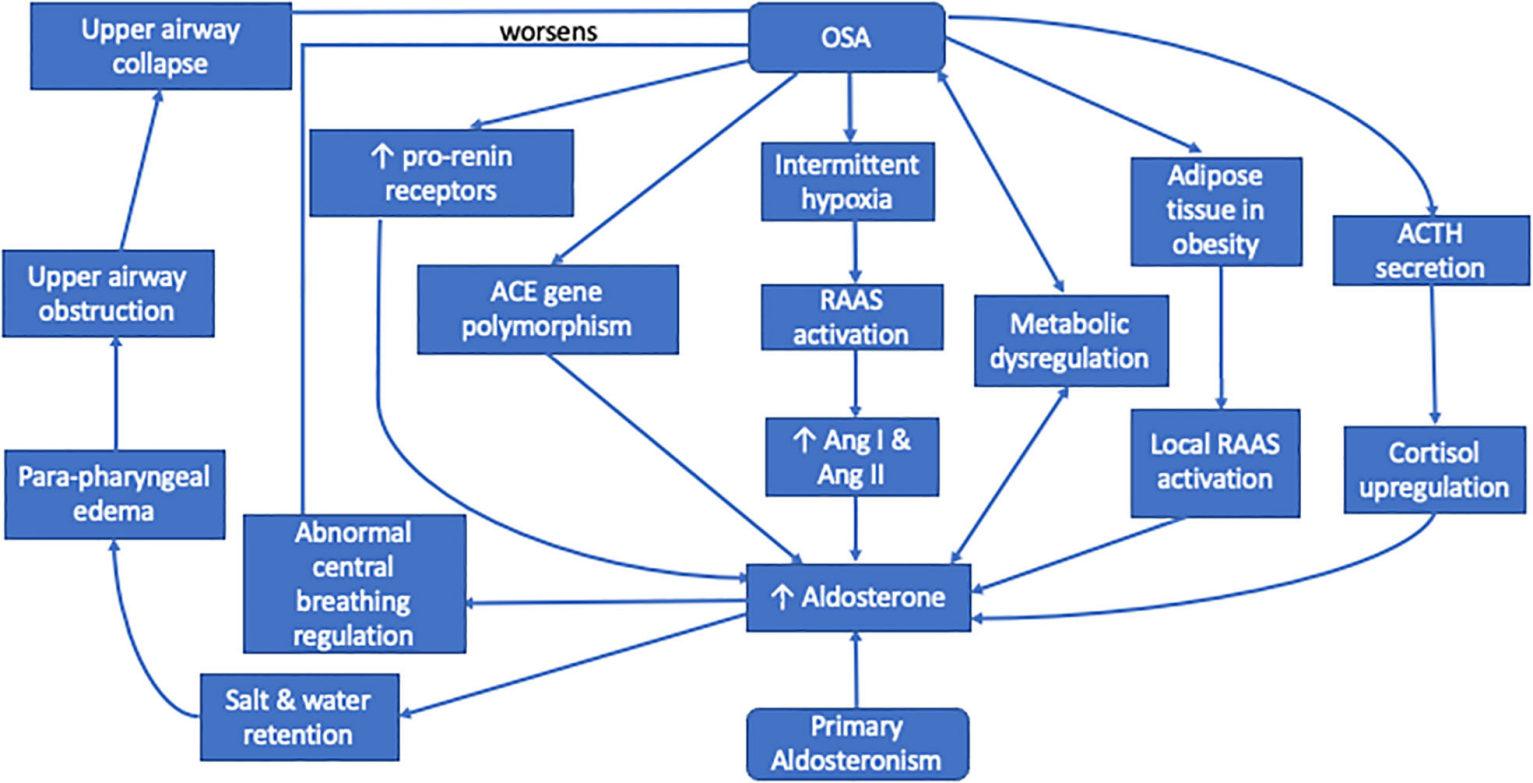
Relationship between primary aldosteronism and obstructive sleep apnea.

The proposed mechanisms of this bi-directional relationship which are understood so far are summarized in **Figure 1**.

## Research gap and future direction

Despite the studies summarized above depicting the relationship between PA, RAAS and OSA, there remains gaps in knowledge with regards to the associations among these entities. Given that clear relationships between PA and OSA have yet to be established, there is a need for further larger prospective studies to examine these links, especially among patients with hypertension, to determine if OSA is truly more prevalent among patients with PA. Likewise, large scale studies to screen the RAAS hormones among patients with OSA are needed to elucidate the true prevalence of PA among this cohort of patients. Risk factors for these patients to have co-existent PA and OSA should be determined to enable clinicians to screen more effectively for the presence of these disorders in high risk patients.

Furthermore, as data of a few studies point toward a likelihood of adipocyte-derived factor releasing adipokines and free fatty acids that could stimulate aldosterone secretion from the adrenocortical cells, independent from the systemic RAAS circulation, studies which examine this local RAAS effect on patients with OSA can further contribute to the knowledge of the relationship between PA and OSA.

It is shown that ACE enzyme is involved in RAAS regulation and ACE gene I/D polymorphism may play a role in hypertension development. As genotype distribution could be contributed by ethnic differences, studies examining ethnic factors can further explain the mechanisms of association between PA and OSA. This might lead to exploration of the role of ethnicity in the complex relationship between PA and OSA, including diagnosis and response to treatment. For example, those with ACE gene polymorphism may respond better to anti-aldosterone treatment for hypertension or blockade of different aspects of the RAAS, hence may benefit more from this class of treatment. Some of the studies have also demonstrated the association between PA and OSA to be more prevalent in specific phenotype with male predominance. Hence studies examining gender differences of this relationship may provide further insights in this field.

Data on the effect of treatment, whether CPAP on RAAS or PA-directed treatment on OSA severity, is substantially insufficient to date. This is especially true for long term outcomes of these treatment, not only on the disease per se, but on cardiovascular outcomes and reduction of target organ damage. This is an important aspect to be explored as it can lead to targeted therapy, which can be beneficial in this cohort of patients. The use of MR antagonists, ACE inhibitor or angiotensin receptor blockers remains low and clinical trials exploring the effect of these treatment are essential towards optimal blood pressure control.

In clinical context, for patients who are confirmed to have PA, OSA screening needs to be considered especially among males and those who are older, with larger neck circumference, greater abdominal obesity, higher BMI and worse metabolic profile. On the other hand, among patients with confirmed OSA, PA should be screened especially if blood pressure is ≥150/100mmHg, or in the presence of resistant hypertension or hypokalemia.

The evidence presented herein further underscores the necessity of early recognition and diagnosis of PA in patients with OSA and hypertension, as well as OSA in patients with PA, in line with the current Endocrine Society Guideline recommendations.

## Conclusion

Current evidence suggests a bi-directional relationship between PA and OSA *via* aldosterone-induced worsening of OSA and OSA-associated dysregulation of RAAS. The beneficial effect seen with treatment of OSA on RAAS as well as PA-directed treatment on OSA severity further supports the role of RAAS-driven pathogenesis in worsening of OSA, as well as the role of OSA-driven pathogenesis in worsening of PA. Nevertheless, given that clear relationship between PA and OSA has yet to be established, there is a need for further studies to examine this link, particularly the role of genotypic and phenotypic relationship, as well as long term beneficial effects of PA-directed therapy in OSA and vice versa.

## Author contributions

HL conceived, designed and drafted the work. NS revised it critically for important intellectual content. All authors contributed to the article and approved the submitted version.

## Funding

The authors received funding from Malaysian Ministry of Higher Education Fundamental Research Grant Scheme (FRGS/1/2021/SKK01/UNIMAS/02/1) and the National University of Malaysia (UKM) grant (FF-2022-066).

## Conflict of interest

The authors declare that the research was conducted in the absence of any commercial or financial relationships that could be construed as a potential conflict of interest.

## Publisher’s note

All claims expressed in this article are solely those of the authors and do not necessarily represent those of their affiliated organizations, or those of the publisher, the editors and the reviewers. Any product that may be evaluated in this article, or claim that may be made by its manufacturer, is not guaranteed or endorsed by the publisher.
